# A Cross-Sectional Study of the Psychological Needs of Adults Living with Cystic Fibrosis

**DOI:** 10.1371/journal.pone.0127944

**Published:** 2015-06-23

**Authors:** Smita Pakhale, Justine Baron, Michael Armstrong, Georgio Tasca, Ena Gaudet, Shawn Aaron, William Cameron, Louise Balfour

**Affiliations:** 1 Ottawa Hospital Research Institute, 501 Smyth Road, K1H 8 M5, Ontario, Ottawa, Canada; 2 The Ottawa Hospital, 501 Smyth Road, K1H 8 M, Ontario, Ottawa, Canada; 3 The University of Ottawa, 451 Smyth Road, K1H 8 M5, Ontario, Ottawa, Canada; 4 Division of Respiratory Medicine, The Ottawa Hospital, 501 Smyth Road, K1H 8 L6, Ottawa, Canada; University of Western Australia, AUSTRALIA

## Abstract

**Background:**

Depression and anxiety are prevalent in people with cystic fibrosis (CF), yet psychological services are rarely accessible in CF clinics. This cross-sectional single center study reports on a psychological needs assessment of people with CF.

**Methods:**

We asked adults attending a CF clinic, without integrated psychological services, to complete a psychological needs assessment survey that included items on: a) past access to psychological services (via a CF referral service), b) concerns relevant to discuss with a psychologist, and c) their likelihood of accessing psychological services if available at the CF clinic, and standardized measures of depression (CES-D) and anxiety (GAD-7).

**Results:**

We enrolled 49 participants and 45 (91.8%) completed the survey. Forty percent reported elevated symptoms of depression and 13% had elevated anxiety. A majority of individuals (72.2% and 83.3%, respectively) indicated they would be likely to use psychological services, if available at the clinic. Concerns considered most relevant to discuss with a psychologist were: 1) worries (51.1%), 2) mood (44.4%), 3) life stress (46.6%), 4) adjustment to CF (42.2%), 5) life transitions (42.2%) and 6) quality of life (42.2%).

**Conclusions:**

This study highlights the rationale for screening adults with CF for depression and anxiety, and to facilitate provision of psychological services and preventative mental health interventions as an integral component of multi-disciplinary CF care.

## Introduction

Cystic Fibrosis (CF) is the most common fatal genetic disease affecting young adults and children in Canada [1]. Progressive lung disease, pancreatic insufficiency leading to fat malabsorption and fat soluble vitamin deficiencies, CF-related diabetes, bone disease, and male infertility are common CF presentations. Common CF symptoms include a chronic and productive cough, shortness of breath, weight loss or failure to thrive, and frequent lung infections requiring repeated antibiotics and/or hospitalizations. Improvements in CF therapies over the past 50 years have increased the median survival from 6 to 49.7 years in Canada and, as a result, over 60% of people with CF are currently adults [2]. Despite these advances, living with CF involves many challenges including complex, time-consuming treatments [3,4] that may take up to 108 minutes per day [5]. A study that examined treatment burden over time in over 7,000 people with CF found that treatment complexity increased over 3 years in all age groups (6–13, 14–17, and 18+ years) and was greatest for adults [4]. In addition, people of all ages living with CF may experience chronic pain—especially in the chest and abdomen [6], and problems with sexuality, relationships, and autonomy [7].

Complex ongoing care and treatment, lifelong symptoms, and other peoples’ perceived reactions to CF may result in people with CF feeling stigmatized[8,9] [8,9]. A subset of individuals with CF experience significant psychological distress and experience negative emotions, poor self-esteem, helplessness, and depressive symptoms [10]. The largest international study on the prevalence of depression and anxiety in people living with CF [11] found that 19% and 32% of adults reported elevated symptoms of depression and anxiety, respectively. Fewer studies have used depression and anxiety screening tools to examine the prevalence of elevated symptoms of depression and anxiety in the general population. Canadian and Swedish studies have done this, and the prevalence of elevated symptoms of depression and anxiety was found to be 15–17.2% and14.7%, respectively [12,13].

Psychological distress has been associated with CF symptoms [14], functional impairment [14], quality of life [15,16], life satisfaction [17], adherence to treatment [18], pulmonary function [16,19], and health care utilization and costs [20]. Psychosocial interventions for people with chronic health conditions, such as cancer and heart disease, have been found to improve psychological outcomes [21]. In CF however, psychological interventions to improve depression and anxiety have received inadequate research attention despite their clinical relevance [22]. Recently published European Cystic Fibrosis Society (ECFS) Standards of Care support the inclusion of a clinical psychologist in each CF care team [23]. These guidelines include recommendations on the key components of a well-functioning, comprehensive, multi-disciplinary CF team, and on the issues a psychologist can address in this population [24].

Goldbeck and colleagues suggest that the lack of research on the effectiveness of psychological interventions in people living with CF could imply that the psychological needs of people with CF are not met [22]. Some of the unmet needs of people living with CF have been highlighted in research [25,26] but these studies have focused on needs related to disease information, and end- of-life care. Psychological needs in people living with cancer have been assessed [27,28], but no such assessment has been conducted in people with CF. Patient needs assessments constitute a key step towards improving quality of care, and achieving greater patient satisfaction [28]. Needs assessments evaluate the gap between patients’ experiences of the services provided and their perceived needs. They also help identify those needs that may not always be successfully communicated to health care professionals [28]. As such, a needs assessment provides an indication of the magnitude of patients’ needs and of the required resources to address them [27]. In addition to informing clinical decision-making and improving patient-doctor communication, needs assessments can help guide care planning by prioritizing service needs, and identifying groups of patients with greater needs [27,28].

Given the importance of needs assessments in healthcare and the greater prevalence of elevated symptoms of depression and anxiety in people with CF, the objective of the current study was to fill a gap in the literature by assessing the psychological needs of adults with CF attending a CF clinic. This multidisciplinary clinic included a CF physician, a social worker, a nurse coordinator, a dietician, a physiotherapist, and access to a psychologist or psychiatrist upon referral to private practice clinicians. We aimed to examine: 1) the extent to which adults with CF access psychological services as part of their CF treatment and care, 2) the likelihood of accessing psychological services if they were available at the CF clinic, and the concerns considered for discussion with a psychologist 3) the likelihood that adults screened positively for depression or anxiety would utilize psychological services if they were available at the clinic. This was an exploratory study with a small sample size, therefore no a priori hypotheses were formulated.

## Materials and Methods

This study comprised part of a larger research project entitled ‘The Ottawa Cystic Fibrosis Treatment Knowledge and Adherence Program’. The Ottawa Health Science Network Research Ethics Board approved this project and all participants provided signed consent.

### Participants

All eligible adults with CF attending a routine visit at the CF clinic between May and July 2011 were approached. Eligible people included adults who were able to provide consent and were expected to continue receiving care at the CF clinic for 1 year. Participants were excluded if they were likely to have a life expectancy of less than one year as per guidelines [29], or if they were lung transplant recipients.

### Recruitment procedures

A member of the research team screened and approached eligible adults in the CF clinic. Following enrolment into the study, participants were asked to complete a questionnaire before their appointment. Medical staff were not present while participants completed their questionnaire.

### Measures

To our knowledge, there is no psychometrically valid psychological needs assessment tool designed for use in people with CF. This psychological needs assessment was conducted in line with the World Health Organization (WHO) guidelines [30]. These guidelines state that an inquiry about the need for specific services, and the necessary capacity, should include: 1) a comprehensive clinical assessment of a sample of individuals, 2) reference to specific types of services that could be made available, 3) the collection of relevant socio-demographic data, and 4) some assurance that those who need the services listed would actually make use of them if the services were made available.

#### Socio-demographics

Our study questionnaire collected data on age, gender, income, educational level, and employment status. Medical data on Body Mass Index (BMI) and Forced Expiratory Volume in 1 second (FEV_1_% predicted) were collected from medical charts.

#### Symptoms of depression

We measured symptoms of depression using the Center for Epidemiologic Studies Depression Scale (CES-D) [31] on which scores range from 0–60. This scale is routinely used in health care settings [32] and has acceptable psychometric properties, with an internal consistency above α = 0.84, test-retest reliability of *r* = 0.40 or above, and moderate convergent validity (*r* = 0.44 or above with other validated depression scales). Scores ≥ 16 are considered to be indicative of elevated (‘mild’ or ‘significant’) symptoms of depression. The sensitivity and specificity of this cut-off in a medically ill population have been found to be 73% and 100%, respectively [33]. In this study, internal consistency of the CES-D was α = 0.90.

#### Symptoms of anxiety

We measured symptoms of anxiety using the Generalised Anxiety Disorder (GAD-7) scale [34] on which scores range from 0–21. The GAD-7 is routinely used in health care settings [35] and has been found to have adequate psychometric properties, with an excellent internal consistency (α = 0.92), good test-retest reliability (r = 0.83), and convergent validity with other validated anxiety scales such as the Beck Anxiety Inventory (r = 0.72) [34]. In this study, the internal consistency of the GAD-7 was α = 0.88. With regards to diagnostic validity, the cut-off point of 10 used in this study is associated with high sensitivity (89%) and specificity (82%) [34].

#### Psychological needs assessment items

These items have been provided in S1 Appendix. They were inspired by our previous experience in needs assessment research [36] and, for item 3 below, by a combination of earlier work on the needs of cancer patients [27], a review of the psychological issues in the lives of patients with CF, and the issues raised by our CF patients in focus group discussions conducted before the study began[9].

Three psychological needs assessment items were developed to enquire about:

1. Past access to psychological services as part of CF treatment and care (‘Currently, how often have you been able to access psychological services at The Ottawa Hospital for your CF treatment care?’ Response options were ‘Never’, ‘Rarely’, ‘Occasionally’, and ‘Often’)
2. The likelihood of accessing these services if they were made available at the CF clinic (‘If made available, how likely would you be to access psychological services at our CF clinic?’ Response options were ‘Very unlikely’, ‘Unlikely’, ‘Likely’, and ‘Very likely’)
3. The CF-related concerns patients felt they needed to talk about with a psychologist (‘If psychological services were easily available and dedicated to patients in our CF clinic as part of our multi-disciplinary team, which of the following concerns would you see a psychologist for (check all that apply)?’). A list of possible concerns was made available, and are presented in the results section of this manuscript. We gave participants additional space to write down any ‘Other’ concern they felt they needed to discuss with a psychologist.

### Data analysis

We used standard descriptive statistics to summarize the characteristics of the sample. We used Fisher Exact tests to examine associations between CES-D and GAD-7 categories (presence or absence of depressive and anxiety symptoms), past access to psychological services, and likelihood of accessing psychological services if available at the CF clinic. To avoid type I error from multiple comparisons, we used Holm’s sequential Bonferroni adjustment [37]. For each statistical test, we have reported an effect size (Phi Coefficient, φ).

## Results

We enrolled 49 (94%) of the 52 adults with CF approached to participate in the study. Four (8%) failed to return completed questionnaires. Characteristics of the 45 remaining participants are presented in Table 1 (see S1 Dataset). Participants’ ages ranged from 18 to 67 years, and the majority were Caucasian. Mean FEV_1_% predicted was indicative of moderate lung disease, and on average study participants had an adequate BMI (24kg/m^2^). Approximately half of the sample had attended University, were employed full-time, and lived with a spouse or partner.

**Table 1:** Characteristics of study participants (n = 45)*.

Variables	Study participants	Variables	Study participants
**Age (mean ±SD)**	30.7 ±10.8	**Relationship status (n,%)**	
**Female gender (n,%)**	19 (42.2)	Single	17 (37.8)
**Caucasian (n,%)**	42 (93.3)	Married/steady partner	27 (60.0)
**Education (n,%)**		Divorced	1 (2.2)
Secondary	9 (20.0)	**Depression (CES-D) (n, %)**	
Some university/college	10 (22.2)	<16	27 (60)
University or more	26 (57.8)	≥16	18 (40)
**Employment status (n,%)**		**Anxiety (GAD) (n, %)**	
Full-time	22 (48.9)	<10	38 (84.4)
Part-time	5 (11.1)	≥10	6 (13.3)
Disability	7 (15.6)	**FEV_1_%predicted** (n = 43)	54.7 (19.9)
Unemployed/retired/other	11 (24.4)	**BMI** (n = 43)	23.4 (4.0)
**Living status (n,%)**			
Alone	6 (13.3)		
Not alone	39 (86.6)		

*Unless indicated otherwise

### Prevalence of elevated symptoms of depression and anxiety

In total, 18 (40%, 95% Confidence Interval [CI] 25.7%-54.3%) participants reported CES-D scores indicative of ‘mild’ or ‘significant’ depression (scores ≥16). Six (13.3%, 95% CI 3.4%-23.3%) participants reported elevated symptoms of anxiety, and six (13.3%, 95% CI 3.4%- 23.3%) participants reported both elevated symptoms of depression and elevated symptoms of anxiety.

### Responses to psychological needs assessment items

Responses to the psychological needs assessment items are presented in Tables 2 and 3. Proportions presented are based on the sample size of each group. There was 2.2% missing data for the GAD-7 and the same proportion of missing data on each of the 3 psychological needs assessment items (see legend in Tables 2 and 3). This explains why some proportions do not add up to 100%.

**Table 2:** Past access to psychological services as part of CF care and likelihood of accessing psychological services if made available.

	Total(n = 45)	CES-D<16(n = 27)	CES-D≥16(n = 18)	*P* *	GAD-7<10(n = 38)	GAD-7≥10(n = 6)	*p*
Past access to psychological services for CF (n,%)^†^				0.723			0.325
Never or Rarely	31(68.9)	18(40)	13(28.9)		28 (62.2)	3(6.7)	
Occasionally or Often	13(28.9)	9 (20)	4(8.9)		9(20)	3(6.7)	
Likelihood of accessing psychological services (n,%)^β^				**0.015**			**0.051**
Unlikely	21(46.6)	17(63.0)	4(22.2)		20(52.6)	0(0)	
Likely	23(51.1)	10(37.0)	13(72.2)		18(47.4)	5(83.3)	

*Note*: n = 45 participants completed the CES-D and n = 44 completed the GAD7).

*P-values displayed are unadjusted. None of the adjusted (Holm’s) p-values were significant.

In bold: p-values for which effect sizes (Phi coefficient) were moderate (≥ 0.30).

^†^ Responses to this item were collapsed into two groups (‘Never or Rarely’ and ‘Occasionally or Often’) given the distribution of the data.

^β^ Responses to this item were collapsed into two groups (‘Likely’/ ‘Unlikely’) given the distribution of the data. Data was missing for one participant on this item.

**Table 3:** Concerns considered relevant for discussion with a psychologist if psychological services were made available at the CF clinic.

Concerns	Total (n = 45)(n,%)	CES-D <16 (n = 27) (n,%)	CESD≥16(n = 18) (n,%)	GAD-7<10(n = 38) (n,%)	GAD-7≥10(n = 6)(n,%)
Mood	20(44.4)	7(25.9)	13(72.2)	14(36.8)	5(83.3)
Relationships	15(33.3)	6(22.2)	9(50.0)	10(26.3)	4(66.7)
Grief/loss	14(31.1)	7(25.9)	7(38.9)	12(31.6)	1(16.7)
Life transitions	19(42.2)	10(37.0)	9(50.0)	16(42.1)	2(33.3)
Quality of life	19(42.2)	10(37.0)	9(50.0)	12(31.6)	6(100)
Pain management	7(15.5)	3(11.1)	4(22.2)	6(15.8)	0(0)
Worries	23(51.1)	11(40.7)	12(6.7)	16(42.1)	6(100)
Body image	7(15.5)	3(11.1)	4(22.2)	5(13.2)	1(16.7)
Self-esteem	10(22.2)	4(14.8)	6(33.3)	7(18.4)	2(33.3)
Life stress	21(46.6)	8(29.6)	13(72.2)	14(36.8)	6(100)
Life/death/existentiality	15(33.3)	7(25.9)	8(44.4)	11(28.9)	3(50)
Neurocognitive difficulties	1(2.2)	1(3.7)	0(0)	0(0)	0(0)
CF adherence	11(24.4)	6(22.2)	5(27.8)	9(23.7)	1(16.7)
Work/life balance	16(35.6)	9(33.3)	7(25.9)	12(31.6)	3(50)
Stigma/Disclosure	10(22.2)	5(18.5)	5(27.8)	7(18.4)	2(33.3)
Traumatic experiences	6(13.3)	2(7.4)	4(22.2)	4(10.5)	1(16.7)
Adjustment to CF	19(42.2)	11(40.7)	8(44.4)	17(44.7)	1(16.7)

*Note*: One participant did not complete this section of the questionnaire

### Past access to psychological services and relationship with CES-D and GAD-7 scores

Participants’ responses to the item on past access to psychological services as part of CF care, and to the item on likelihood of accessing psychological services if available, are presented in Table 2. Responses are presented for the study sample as a whole, and according to depression and anxiety cut-off scores. The majority (68.9%) of participants had ‘*never*’ or ‘*rarely*’ accessed psychological services, of which 28.9% reported elevated depressive symptoms. Past access to psychological services in CF care was not significantly related to participants’ levels of depressive symptoms (unadjusted p = 0.753, φ = 0.11) or anxiety (unadjusted p = 0.325, φ = 0.20), and the effect sizes were small.

### Likelihood of accessing psychological services, and relationship with CES-D and GAD-7 scores

Over half (51.1%) of the study sample reported that it was likely they would access psychological services if available at their CF clinic. Over 70% of participants reporting elevated symptoms of depression stated that they would access such psychological services. The majority (63%) of those with low symptoms of depression reported that it was unlikely they would access psychological services. The association between depressive symptoms and likelihood to use psychological services if available approached significance (unadjusted *p* = 0.015, which is greater than Holm’s adjusted *p* = 0.013). The effect size was medium (φ = 0.384), suggesting the statistical non-significance of the association is probably related to low power, rather than to a finding that is not likely in the population.

A similar pattern in the relationship between anxiety and likelihood to access psychological services was observed, although this was not statistically significant (unadjusted *p* = 0.051). The effect size however was medium (φ = 0.338). In total, 83.3% of participants reporting elevated symptoms of anxiety indicated they would be likely to access psychological services if available. Surprisingly, almost half (47.7%) of those reporting low anxiety symptoms reported that they would be likely to use psychological services if available.

### Concerns participants were interested in discussing with a psychologist

We asked participants to indicate the type of concerns they would be interested in discussing with a psychologist if psychological services were available at their CF clinic (the list of concerns and participants’ responses are presented in Table 3). Table 3 presents participants’ responses in the study sample as a whole, and in relation to participants’ CES-D and GAD-7 scores. Small sample size precluded us from conducting statistical tests to examine associations between concerns considered relevant by participants, and depression and anxiety scores.

Participants’ responses show that more than 40% of individuals were interested in seeing a psychologist to discuss worries, life stress, mood, adjustment to CF-related issues/milestones, life transitions, and quality of life. The proportion of participants interested in discussing pain management, body image, neurocognitive difficulties, and traumatic experiences, was less than 16%. Only 3 participants entered a concern in the ‘Other’ response option. The ‘Other’ concerns entered were ‘expectations with regards to hospital appointments’, ‘help to quiet the mind’, and ‘marital issues’.

## Discussion

The aim of the current study was to assess the psychological needs of adults living with CF. As per the WHO [30] recommendations, we included a clinical assessment of depressive symptoms and anxiety, and examined the relationship between scores on these clinical assessments and likelihood to use psychological services if available at the clinic.

The participation rate in the current study was 94%. This suggests that the study sample, although small, may be representative of the adult population living with CF in Ottawa. First, several observations can be made about the prevalence of symptoms of depression and anxiety in the study sample. In our study, 40% of the sample reported elevated symptoms of depression (CES-D scores≥ 16). This prevalence is higher than in the general population [38], and slightly greater compared to a large study in which 29% of adults with CF reported elevated depressive symptoms on the CES-D [11]. Prevalence rates in women tend to be higher compared to men [39], but the current study sample was fairly balanced with regards to sex (42.2% females). Importantly, 20% of study participants reported low levels of depression and regular (‘*occasionally*’ or ‘*often*’) past access to psychological services. A positive interpretation of this, is that accessing psychological services had a protective effect on participants’ symptoms of depression.

In terms of anxiety scores, a total of 13% of our study sample reported elevated symptoms of anxiety on the GAD-7. This prevalence rate is lower than the rate reported in a large sample of adults with CF (32%). On the other hand, it is above the prevalence of elevated anxiety symptoms (5%) in another study on 153 adults with CF [40], and above the prevalence of Generalized Anxiety Disorder ([GAD], 1.1%) in the general Canadian population [41]. Discrepancies in the prevalence of symptoms of anxiety in the studies cited above may be related to the assessment tools or clinical cut-off points used. One study relied on the Hospital Anxiety and Depression Scale (HADS) [41], while the other used the GAD-7 and the same clinical cut-off as in our study [40]. Finally, anxiety-depression comorbidity is observed in the general population [42]. Although prevalence of this comorbidity has not been systematically investigated in samples of adults with CF [14], the current study suggests that 13% of adults with CF may suffer from both; this compares to 14% in a sample of over 4,000 adults with CF from nine countries [11].

Our study data suggests that approximately 30% of those experiencing elevated depression symptoms in the sample had ‘*never*’ or ‘*rarely*’ accessed psychological services. There were fewer participants with elevated symptoms of anxiety in the study sample, and half of them had ‘*never*’ or ‘*rarely*’ accessed psychological services. It is it not uncommon for people who experience symptoms of depression or anxiety to not receive the psychological support they need. In an Australian study, 75% of clinically depressed cancer patients did not receive psychological treatment, despite the availability of counseling and support groups [43]. Two US-based studies showed that the mental health care costs and visits for patients with and without depression were similar, despite differences being significant for other health care services [44,45]. In another study, a large proportion (64%) of University students with major depression were not receiving therapy, despite having health insurance and free access to short-term therapy from campus providers [46]. In addition, people suffering from anxiety may be less likely to use mental health services than people with mood disorders [47]. These data suggest that problems in accessing psychological treatment are not restricted to CF and Canadian populations. If psychological services are available at a CF clinic, other interventions promoting their use should be delivered in parallel.

The majority (72%) of participants reporting elevated symptoms of depression and anxiety (83.3%) reported that they would be likely to use psychological services if they were made available at their CF clinic. A large proportion of those who did not report symptoms of depression (63%) or anxiety (52.6%) showed interest in using psychological services. As Oxley and colleagues state [48], the range of psychological stresses and difficulties people with CF experience may not be identified by measures of depression and anxiety. Participants in our study were not only interested in discussing worries (51.1%) and mood (44.4%) with a psychologist, they were also interested in discussing life transitions (42.2%), quality of life (42.2%), life stress (46.6%), and adjustment to CF (42.2%). As such, a first recommendation supported by existing guidelines [49] would be to implement annual routine psychological screening of patients with CF. This would help identify patients at risk of depression and anxiety, and allow them to be given priority access to a psychologist. To be effective, screening strategies need to account for barriers to implementation identified in recent work [50], the most commonly cited by CF health care professionals being limited staff time, limited personnel, and lack of qualified personnel to provide referrals or interventions. The high proportion of participants in our study with low symptoms of depression or anxiety that showed interest in seeing a psychologist underlines the need to deliver the preventative and supportive interventions recommended by the Cystic Fibrosis Foundation (CFF) and ESCF [51]. According to these guidelines on mental health, these interventions could include training in stress management, development of coping skills aligned with appropriate developmental stages and life disease events, and behavioural approaches to reduce the risk of distress, particularly for those undergoing medical procedures.

The high proportion of participants in our study that were interested in discussing concerns with a psychologist may also be related to a self-selection bias. This remains unlikely however, as the psychological needs assessment reported in this paper was part of a larger research program on treatment adherence. Another explanation is related to the diagnostic performance of the CES-D and GAD-7. This remains unlikely as they have been shown to be good diagnostic measures and are commonly used in CF research [11,40,52]. Our study was designed prior to the CFF-ESCF guidelines recommending the use of the Patient Health Questionnaire (PHQ-9) and of the GAD-7 to screen for depression and anxiety. Further CF research on mental health may benefit from using the PHQ-9 over the CES-D. The PHQ-9 is advantageous for several reasons: 1) it is based on the Diagnostic and Statistical Manual of Mental Disorders (DSM-IV) criteria, 2) it is increasingly used across patient groups in research and health care settings, 3) it is easy to score, 4) it has been translated for use in many countries, 5) it is half the length of the CES-D but has comparable sensitivity and specificity, and 6) it is more appropriate than the CES-D to evaluate treatment response as there is more information on its sensitivity to change.

There is little research on the effectiveness of psychological interventions in patients with CF [22,48]. As was underlined by Oxley and colleagues [48], there is no reason people with CF would not benefit from such interventions when they have been shown to be effective in other populations. Several interventions have been found to improve depression and anxiety in people with other medical conditions [21]. Offering psychological interventions to patients with CF may also improve the effectiveness of medical co-interventions [53]. People affected with other life-threatening conditions, such as cancer, have also reported that their psychological needs are not well-addressed by health care [27,28], and a call for general medical guidelines to specify treatment recommendations for people suffering from depression has been made [54].

This study is the first description of the psychological needs of adults with CF attending a Canadian adult multidisciplinary CF clinic where a social worker is available, but access to a clinical psychologist is only available through a private practice referral system. This situation is common in CF clinics across Canada. In 2011, Cystic Fibrosis Canada released a summary of the services available at the 42 CF accredited clinics in Canada [55]. In this document, the services offered by psychologists and psychiatrists are presented together despite the important differences in roles between these professions. The data do however provide some overall indication of the level of access to mental health services in Canadian CF clinics. Only 16 (38.1%) of 42 CF clinics had a psychologist/psychiatrist integrated to the CF multidisciplinary care team, and community private practice referrals were available in 20 CF clinics (47.6%). In 3 of the Canadian CF clinics (7.1%), there was no psychologist/psychiatrist integrated to the CF team, and no referral system in place.

European countries have agreed that there is a need for CF teams to include a clinical psychologist, and this is now part of their standards of care [23]. As such, many Canadian CF clinics do not meet international standards for CF care. Referral systems are sometimes in place but they have several disadvantages. First, they are often associated with excessively long waiting times, sometimes up to 12 months (personal communication with an Adult Cystic Fibrosis Nurse at the Royal University Hospital, Saskatoon, October 2012). A second disadvantage of referral systems is that it is more difficult to create and maintain a working partnership between the CF care team and the psychologist/psychiatrist, especially when these services are offered in different locations. This is important, as an integrated team approach is key in the care of people with chronic health conditions [48]. A third disadvantage of referral systems relates to the reimbursement scheme associated with these referrals in Canada. Psychological services offered in hospital-based CF clinics are covered by universal provincial health insurance plans; referrals to private practice psychologists outside the CF clinic team are not. The majority of people with CF are unlikely to be able to afford expensive private practice psychological services, or to have access to extended employee health benefits that could help cover the costs (40% of participants in our study were unemployed or on disability). The limited availability of publicly funded psychological services in health settings, and the lack of affordability of private services, was underlined by the Canadian Government in 2004 [56], yet this has not translated to improvements in Canadian CF care.

This study has several limitations. The data was collected at a single Canadian site, and therefore the findings may not be generalizable to other populations with CF internationally. CF clinics in European countries are more likely to include a psychologist [50], and some of our findings may be less applicable to them. Nonetheless, 54% of adults with CF surveyed about psychosocial provision in CF centres in the UK reported that their CF centre did not have a clinical psychologist. In addition, 20% of those who accessed a clinical psychologist did this through a referral system [57]. These numbers suggest some similarities between the UK and the Canadian system. Only 14% of adults with CF in the UK reported waiting more than one month for an appointment with a clinical psychologist however, which suggests the UK may be better equipped to address the demand. Countries differ with respect for example to their health care delivery and payment systems, practice structures, training (etc.). As such, different national implementation strategies will be required to integrate mental health into CF care [50].

Another limitation of this study is that participants were adults, and a paediatric population may have different needs and preferences with regards to accessing psychological services. We chose to use self-report measures of mood that have undergone rigorous psychometric testing, and that are commonly used in heath settings. We do however recognize that as self-report measures, they have their limitations, and they are not as comprehensive and reliable as a diagnostic interview. We also recognize that the sample size remains small for a survey study, and it is for this reason that we limited the number of statistical tests performed. A multicenter design that includes a control group for comparison and that involves a larger sample size is recommended for future research in this area.

## Conclusion

Our study highlights the psychological needs of adults with CF, with regards to both prevention and treatment. Despite the availability of mental health services in some of the CF care teams across the country, psychological services are not available to patients with CF in the majority of Canadian CF clinics. This means patients with CF are receiving sub-standard care. There is an important need to continue to promote an integrated team approach to CF care, and to underline the need for multidisciplinary CF care teams to include a clinical psychologist.

## Supporting Information

S1 Appendix Psychological needs assessment items. (DOCX)

S1 Dataset Study data. (XLS)
