# Supplementary material for: A Cross-Sectional Study of the Psychological Needs of Adults Living with Cystic Fibrosis
Source: PLoS One. 2015 Jun 23;10(6):e0127944. doi: 10.1371/journal.pone.0127944 (PMC4478009; doi:10.1371/journal.pone.0127944)
Supplement: S1 Appendix — (DOCX) [file pone.0127944.s001.docx]

**S1 Appendix. Psychological Needs Assessment Survey**

**Psychological Needs Assessment Survey**

Currently, people with cystic fibrosis attending this clinic can be referred by members of our CF care team to psychological services outside The Ottawa Hospital. We would like to find out how often you have accessed these psychological services through our referral system, whether there are specific concerns you would like to discuss with a psychologist, and whether you would be likely to see a psychologist if one was available at our CF clinic. Your answers to the following questions can help us improve the services this CF clinic can offer you.

1. **Currently, how often have you been able to access psychological services at the Ottawa Hospital for your CF treatment care?**

❑ ❑ ❑ ❑

Never Rarely Occasionally Often

1. **If psychological services were easily available and dedicated to patients in our CF clinic as part of our multi-disciplinary team, which of the following concerns might you want to be able to see a psychologist for (check all that apply):**

| ❑ Help coping with **mood** (e.g., sadness, loss of interest) | ❑ Help with **worrie**s (e.g., anxiety) | ❑ Coping with **CF Treatment adherence** |
| --- | --- | --- |
| ❑ Help with **interpersonal relationship issues** | ❑ Help with issues related to **body image** | ❑ Managing **work-life balance** |
| ❑ Coping with **grief/loss** | ❑ Improving **self-esteem** | ❑ Help and coping with **Stigma and/or CF Disclosure** |
| ❑ Support for **life transitions** (e.g., employment, independent living, children) | ❑ Managing **life stress** | ❑ Support with issues related to **traumatic experiences** |
| ❑ Improving general **quality of life** | ❑ Coping with issues of **life/death, existential issues** | ❑ **Adjusting to CF** **related issues/milestones** (e.g., lung transplant, new treatments) |
| ❑ Help with **pain management** | ❑ Help with **neuro-cognitive** difficulties |  |
| ❑ **Other concern(s)** (please indicate): **_____________________________________________** | | |

1. **If made available, how likely would you be to access psychological services at our CF clinic?**

❑ ❑ ❑ ❑

Very unlikely Unlikely Likely Very likely
